# Belatacept Rescue Therapy in the Early Period After Simultaneous Kidney-Pancreas Transplantation

**DOI:** 10.3389/ti.2024.12628

**Published:** 2024-04-11

**Authors:** Laure Esposito, Emmanuel Cuellar, Olivier Marion, Arnaud Del Bello, Anne Laure Hebral, Federico Sallusto, Fabrice Muscari, Thomas Prudhomme, Nassim Kamar

**Affiliations:** ^1^ Department of Nephrology and Organ Transplantation, Toulouse University Hospital, Toulouse, France; ^2^ Department of Digestive Surgery, Toulouse University Hospital, Toulouse, France; ^3^ INSERM UMR 1291, Toulouse Institute for Infectious and Inflammatory Diseases (Infinity), Toulouse, France; ^4^ Department of Urology and Renal Transplantation, Toulouse University Hospital, Toulouse, France; ^5^ Université Paul Sabatier, Toulouse, France

**Keywords:** belatacept, kidney transplantation, pancreas transplantation, gastroparesis, delayed graft function

Dear Editors, Belatacept has been used as a rescue therapy in kidney-transplant patients and in a heart-liver-kidney transplant patient with prolonged delayed graft function (DGF) [1, 2]. Although, encouraging results were observed in kidney-transplant patients with preexisting diabetes [3], very few simultaneous kidney-pancreas-transplant (SKPT) patients were given belatacept [4, 5]. Gastroparesis, a common complication in diabetic patients, is a syndrome defined by symptoms and delayed gastric emptying in the absence of mechanical obstruction [6]. Typical symptoms include nausea, vomiting, abdominal pain, and early satiety [6]. Gastroparesis can be responsible at impaired drug absorption, including immunosuppressants [6].

Herein, we report the use of belatacept as rescue therapy in four SKPT because of severe gastroparesis responsible for large tacrolimus trough levels variability (*n* = 3) and/or prolonged delayed graft function (*n* = 2) (Table 1).

**TABLE 1:** Patients’ characteristics and outcome.

	Age at transplantation (years)	Gender	Anti-HLA antibodies/Preformed donor specific antibodies	Time between transplantation and belatacept initiations (days)	Serum creatinine level at the initiation of belatacept (µmol/L)	Duration of belatacept (months)	Serum creatinine level at belatacept stop (µmol/L)	Time between transplantation and last follow-up (months)	Serum creatinine level at last-follow-up (µmol/L)
Patient 1	38	Male	No/No	15	174	1.5	116	110	96
Patient 2	55	Female	Yes/No	23	Dialysis	Ongoing	-	3	150
Patient 3	40	Female	No/No	81	Dialysis	1.5	123	8	100
Patient 4	32	Male	Yes/No	170	269	Ongoing	122	7	122

At transplantation, all patients had been given polyclonal antibodies (Thymoglobulins^®^, Sanofi; 3.75 mg/kg total dose), tacrolimus (Prograf^®^, Astellas Pharma) and mycophenolic acid. Steroids were scheduled to be stopped within the first 10 days after transplantation. Only one patient was maintained on prednisone (5 mg/d) for 3 months.

Since in the BENEFIT phase III trials, an increased risk of acute rejection was observed in belatacept-treated patients compared to those given cyclosporine A-based therapy [7, 8], when belatacept was initiated in our patients, it was given with low-dose tacrolimus, and MPA (500 mg b.i.d that remained unchanged). Belatacept was administrated at the dose of 6 mg/kg at days 0 and 15 and then every 4 weeks. All patients were Epstein Barr Virus IgG positive.

Patient 1 started belatacept at day 15 post-transplantation because of severe gastroparesis, vomiting, tacrolimus malabsorption and large variations of tacrolimus trough levels (Tac C0) that ranged between 3.8 and 52 ng/mL (median = 12) using tacrolimus at 7–10 mg/d. At belatacept initiation, it was at 12.8 ng/mL. When associated to belatacept, Tac C0 was maintained between 4 and 5 ng/mL. Belatacept was stopped 1.5 months later (after 3 doses) when gastrointestinal symptoms had disappeared. After belatacept stop, Tac C0 ranged between 7 and 8 ng/mL using tacrolimus 6 mg/d. Serum creatinine level decreased from 174 μmol/L at belatacept initiation to 116 μmol/L when it was stopped.

Patient 2 had gastroparesis symptoms and prolonged DGF. Belatacept was started at day 23 while she had still had gastrointestinal symptoms and was still requiring dialysis. A kidney allograft biopsy revealed the presence of isolated acute tubular necrosis (ATN). Tac C0 ranged between 4.4 and 12 ng/mL while Tac dose was unchanged (12 mg/d), and was at 11 ng/mL at belatacept initiation. When associated to belatacept, Tac C0 was maintained between 4 and 5 ng/mL. After the initiation of belatacept, gastrointestinal symptoms improved and kidney function recovered. At last follow-up, i.e. 3 months after transplantation, she is still given belatacept-based therapy and her serum creatinine level is at 150 μmol/L.

Patient 3 experienced several complications after transplantation, namely, infections of peripancreatic fluid collections requiring antibiotics and antifungal therapies. She had a prolonged DGF. At day 81, she was still requiring hemodialysis. A kidney allograft biopsy showed isolated severe ATN. Hence, belatacept was started. Tac C0 was at 8 ng/mL. When associated to belatacept, Tac C0 was at 4 ng/mL. Kidney function recovered rapidly and belatacept was stopped after 3 administrations, i.e. 1.5 months after its initiation. After belatacept stop, Tac C0 ranged between 7 and 8 ng/mL. Serum creatinine level had decreased to 123 μmol/L.

Finally, patient 4 presented several episodes of gastrointestinal symptoms attributed to gastroparesis after transplantation. This was associated each time with an impairment of kidney function. At 5 months post-transplantation he was admitted for a severe gastroparesis episodes associated with large Tac C0 variations and acute kidney injury. A kidney allograft biopsy revealed the presence of isolated ATN belatacept was initiated and is still pursued until last follow-up, i.e.,7 months after transplantation. Tac C0 ranged between 5.6 and 18 ng/mL while tacrolimus dose was unchanged (8 mg/d), and was at 11 ng/mL at belatacept initiation. When associated to belatacept, Tac C0 was maintained at 5 ng/mL. Serum creatinine level decreased from 269 at the initiation of belatacept to 122 at last follow-up.

At the initiation of belatacept, only one patient (patient 2) who was receiving parenteral nutrition was still given insulin while all other three patients were insulin-free. At last follow-up, none of the patient was given insulin and c-peptide level was at 3.75 (3.3–4.7) ng/mL. No acute rejection, *de novo* DSA or infection occurred after the initiation of belatacept. BK virus DNAemia was negative in all patients In the 2 patients in whom belatacept was stopped and tacrolimus doses re-increased, no episode of gastrointestinal symptoms occurred after belatacept stop. In the two other patients, it was decided to pursue belatacept and to stop tacrolimus at one-year posttransplant.

In a phase II prospective study, *de novo* SKPT patients were randomized to receive a tacrolimus based immunosuppressive regimen or belatacept and low-dose tacrolimus [5]. At Week 40, in the absence of an history of acute rejection and in patients having stable grafts’ functions, tacrolimus was withdrawn. The biopsy proven acute rejection rates of the pancreas and the kidney were low and similar in both arms before tacrolimus withdrawal in the belatacept arm [5]. However, an increased risk of pancreas rejection was observed during and after tacrolimus withdrawal [5]. The authors concluded that belatacept did not provide sufficient immunosuppression to reliably prevent pancreas rejection in SKPT patients undergoing calcineurin inhibitors withdrawal. Conversely, late conversion to belatacept in SKPT patients was found to be safe [4]. In our report, since the initiation of belatacept was done within the first months after transplantation, we have chosen to maintain a low-dose of tacrolimus in addition to belatacept. This strategy was safe.

Our short case series suggests that in selected SKPT patients with severe gastroparesis responsible for immunosuppressants malabsorption and/or in those presenting a prolonged DGF, a transient or prolonged course of belatacept associated with low-dose tacrolimus can be considered. Further studies including a larger number of patients are required to confirm theses preliminary data.

## Data Availability

The raw data supporting the conclusion of this article will be made available by the authors, without undue reservation.
